# Optimal adaptive control for quantum metrology with time-dependent Hamiltonians

**DOI:** 10.1038/ncomms14695

**Published:** 2017-03-09

**Authors:** Shengshi Pang, Andrew N. Jordan

**Affiliations:** 1 Department of Physics and Astronomy, University of Rochester, Rochester, New York 14627, USA; 2 Center for Coherence and Quantum Optics, University of Rochester, Rochester, New York 14627, USA; 3 Institute for Quantum Studies, Chapman University, 1 University Drive, Orange, California 92866, USA

## Abstract

Quantum metrology has been studied for a wide range of systems with time-independent Hamiltonians. For systems with time-dependent Hamiltonians, however, due to the complexity of dynamics, little has been known about quantum metrology. Here we investigate quantum metrology with time-dependent Hamiltonians to bridge this gap. We obtain the optimal quantum Fisher information for parameters in time-dependent Hamiltonians, and show proper Hamiltonian control is generally necessary to optimize the Fisher information. We derive the optimal Hamiltonian control, which is generally adaptive, and the measurement scheme to attain the optimal Fisher information. In a minimal example of a qubit in a rotating magnetic field, we find a surprising result that the fundamental limit of *T*^2^ time scaling of quantum Fisher information can be broken with time-dependent Hamiltonians, which reaches *T*^4^ in estimating the rotation frequency of the field. We conclude by considering level crossings in the derivatives of the Hamiltonians, and point out additional control is necessary for that case.

## Abstract (web summary)

Quantum metrology investigates the improvement given to precision measurements by exploiting quantum mechanics, but it has been mostly limited to systems with static Hamiltonians. Here the authors study it in the general case of time-varying Hamiltonians, showing that optimizing the quantum Fisher information via quantum control provides an advantage.

Precision measurement has been long pursued due to its vital importance in physics and other sciences. Quantum mechanics supplies this task with two new elements. On one hand, quantum mechanics imposes a fundamental limitation on the precision of measurements, apart from any external noise, the quantum noise^1^, which is rooted in the stochastic nature of quantum measurement and manifested by the Heisenberg uncertainty principle. On the other hand, quantum mechanics also opens new possibilities for improving measurement sensitivities by utilizing non-classical resources, such as quantum entanglement and squeezing^2^. These have given rise to the wide interest in quantum parameter estimation^3^,^4^ and quantum metrology^5^,^6^. Since its birth, quantum metrology has been applied in many areas, ranging from gravitational wave detection^7^,^8^,^9^, quantum clocks^10^,^11^, quantum imaging^12^,^13^,^14^, to optomechanics^15^, quantum biology^16^ and so on. Various quantum correlations have been shown useful for enhancing measurement sensitivities, including spin-squeezed states^17^,^18^,^19^,^20^,^21^,^22^, N00N states^23^,^24^,^25^,^26^,^27^ and so on. Nonlinear interactions have been exploited to break the Heisenberg limit even without entanglement^28^,^29^,^30^,^31^,^32^,^33^,^34^,^35^. For practical applications where disturbance from the environment is inevitable, quantum metrology in open systems has been studied^36^,^37^,^38^,^39^,^40^, and quantum error correction schemes for protecting quantum metrology against noise have been proposed^41^,^42^,^43^,^44^,^45^.

While most previous research on quantum metrology was focused on multiplicative parameters of Hamiltonians, growing attention has recently been drawn to more general parameters of Hamiltonians^46^ or physical dynamics^47^,^48^, such as those of magnetic fields^46^,^49^,^50^,^51^. Interestingly, in contrast to estimation of multiplicative parameters, estimation of general Hamiltonian parameters exhibits distinct characteristics in some aspects, particularly in the time scaling of the Fisher information^46^, and often requires quantum control to gain the highest sensitivity^52^.

While there has been tremendous research devoted to quantum metrology, most of those works were focused on time-independent Hamiltonians, and little has been known when the Hamiltonians are varying with time. (The most relevant work so far to our knowledge includes ref. 53 that uses basis splines to approximate a time-dependent Hamiltonian of a qubit, and ref. 54 that studies the quantum Cramér–Rao bound for a time-varying signal and so on) Nevertheless, in reality, many factors that influence the systems are changing with time, for example, periodic driving fields or fluctuating external noise. In the state-of-the-art field of quantum engineering, fast varying quantum controls are often involved to improve operation fidelity and efficiency. Therefore, the current knowledge about quantum metrology with static Hamiltonians significantly limits application of quantum metrology in broader areas, and the capability of treating time-dependent Hamiltonians is intrinsically necessary for allowing the applicability of quantum metrology in more complex situations.

In this article, we study quantum metrology with time-dependent Hamiltonians to bridge this gap. We obtain the maximum quantum Fisher information for parameters in time-dependent Hamiltonians in general, and show that it is attainable only with proper control on the Hamiltonians generally. The optimal Hamiltonian control and the measurement scheme to achieve the maximum Fisher information are derived. Based on the general results obtained, we surprisingly find that some fundamental limits in quantum metrology with time-independent Hamiltonians can be broken with time-dependent Hamiltonians. In a minimal example of a qubit in a rotating magnetic field, we show that the time-scaling of Fisher information for the rotation frequency of the field can reach *T*^4^ in the presence of the optimal Hamiltonian control, significantly exceeding the traditional limit *T*^2^ with time-independent Hamiltonians. This suggests substantial differences between quantum metrology with time-varying Hamiltonians and with static Hamiltonians. Finally, we consider level crossings in the derivatives of Hamiltonians with respect to the estimated parameters, and show that additional Hamiltonian control is generally necessary to maximize the Fisher information in that case.

## Results

### Quantum parameter estimation

Parameter estimation is an important task in vast areas of sciences, which is to extract the parameter of interest from a distribution of data. The core goal of parameter estimation is to increase the estimation precision. The estimation precision is determined by how well the parameter can be distinguished from a value in the vicinity, which can usually be characterized by the statistical distance between the distributions with neighbouring parameters^55^. The well-known Cramér–Rao bound^56^ shows the universal limit of precision for arbitrary estimation strategies, which indicates that for a parameter *g* in a probability distribution *p*_*g*_(*X*) of some random variable *X*, the mean squared deviation 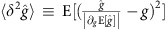 is bounded by


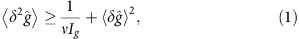


where *ν* is the amount of data, *I*_*g*_ is the Fisher information^57^,


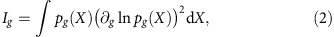


and 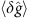 is the mean systematic error. For an unbiased estimation strategy, 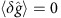. The Cramér–Rao bound can generally be achieved with the maximum likelihood estimation strategy when the number of trials is sufficiently large^57^. In practice, however, due to the finiteness of resource, only a limited number of trials are available usually. For such situations, the Cramér–Rao bound may become loose, and new families of error measures have been proposed to give tighter bounds, for example ref. 58. In this paper, we pursue the ultimate precision limit of quantum metrology with time-dependent Hamiltonians allowed by quantum mechanics, regardless of any practical imperfections like the finiteness of resources or external noise, so the Cramér–Rao bound is the proper measure for the estimation precision.

In the quantum regime of parameter estimation, we are interested in estimating parameters in quantum states. The essence of estimating a parameter in a quantum state is distinguishing the quantum state with the parameter of interest from that state with a slightly deviated parameter. When the quantum state is measured, the parameter in that state controls the probability distribution of the measurement results, and the information about the parameter can be extracted from the measurement results. As there are many different possible measurements on the same quantum state, the Fisher information needs to be maximized over all possible measurements so as to properly quantify the distinguishability of the quantum state with the parameter of interest. It is shown by refs 59, 60 that the maximum Fisher information for a parameter *g* in a quantum state 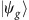 over all possible generalized quantum measurements is


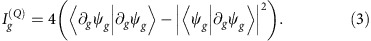


This is called quantum Fisher information, and is closely related to the Bures distance 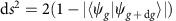 (ref. 61) through 
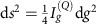
 between two adjacent states 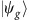 and 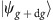, which characterizes the distinguishability between 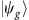 and 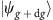.

**Figure 1:**
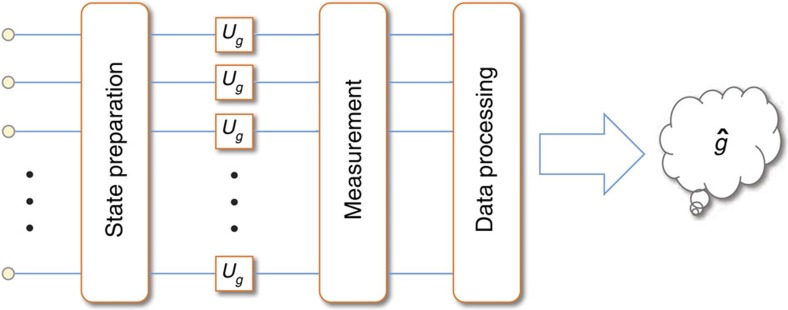
General procedures of quantum metrology. Quantum metrology can generally be decomposed to four steps: preparation of the initial states of the quantum systems, parameter-dependent evolution (*U*_*g*_ in the figure) of the systems, measurements on the final states of the systems, and post processing of the measurement data to extract the parameter. Each node at the left side of the figure represents one quantum system (which can be very general and consist of subsystems). Usually multiple systems are exploited to undergo such a process, and they can be entangled at the preparation step to increase the estimation precision beyond the standard quantum limit, which is the advantage of quantum metrology.

In quantum metrology, the parameters to estimate are usually in Hamiltonians, or more generally, in physical dynamics. The parameters are encoded into quantum states by letting some quantum systems evolve under the Hamiltonians or physical dynamics of interest. The states of the systems acquire the information about the parameters from the evolution. The parameters can then be learned from measurements on the final states of the systems with appropriate processing of the measurement data. A general process of quantum metrology is depicted in Fig. 1.

A simple and widely studied example of quantum metrology is to estimate a multiplicative parameter in a Hamiltonian, say, to estimate *g* in *H*_*g*_=*gH*_0_ (ref. 62), where *H*_0_ is time-independent. In this case, if a quantum systems undergoes the unitary evolution *U*_*g*_=exp(−*igH*_0_*T*) for some time *T*, the quantum Fisher information (3) that determines the estimation precision of *g* is 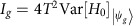, where Var[·] represents variance and 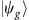 is the final state of the system. A more general case concerns a general parameter in a Hamiltonian^46^. The quantum Fisher information for a general parameter *g* in a Hamiltonian *H*_*g*_ is 
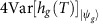
, and 
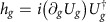
 is the local generator of the parametric translation of *U*_*g*_=exp(−*iH*_*g*_*T*) with respect to *g* (ref. 60).

Compared to classical precision measurements, the advantage of quantum metrology is that non-classical correlations can significantly enhance measurement sensitivities. Various kinds of non-classical correlations have been found useful for improving measurement precision, as reviewed in the introduction. With *N* properly correlated systems, the quantum Fisher information can beat the standard quantum limit and attain the Heisenberg scaling *N*^2^ by appropriate metrological schemes^5^.

### Time-dependent quantum metrology

We now turn to the main topic of this work, quantum metrology with time-dependent Hamiltonians. Our goal is to find the maximum Fisher information for parameters in time-dependent Hamiltonians.

The starting point of quantum metrology with a time-dependent Hamiltonian is similar as with a time-independent Hamiltonian above. A system is initialized in some state 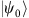 and evolves under the time-dependent Hamiltonian *H*_*g*_(*t*) with *g* as the parameter to estimate, then after an evolution for some time *T*, one measures the final state of the system


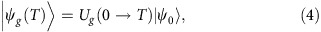


where *U*_*g*_(0→*T*) is the unitary evolution under the Hamiltonian *H*_*g*_(*t*) for time *T*, and estimates *g* from the measurement results, which is just the standard recipe for a general quantum metrology. And the quantum Fisher information of estimating *g* from measuring 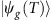 is still determined by equation (3), which can be written as 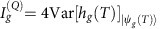, where 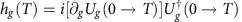.

Everything is similar as before so far, but we can immediately see two major obstacles to deriving the maximum Fisher information. One is that due to the complexity of evolution under a time-dependent Hamiltonian, the unitary evolution *U*_*g*_(0→*T*) is generally difficult to obtain. The other is that even if we can find a solution to *U*_*g*_(0→*T*), it is hard to maximize the Fisher information, since *h*_*g*_(*T*) can be quite complex and the optimization is global involving the whole evolution history of the system for time *T*. To derive the maximum Fisher information for time-dependent Hamiltonians, we need to overcome these obstacles.

For the purpose of convenience, we first reformulate the quantum Fisher information as


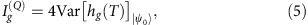


which is dependent on the initial state 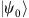 of the system now, and *h*_*g*_(*T*) becomes *i*
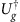
(0→*T*)*δ*_*g*_*U*_*g*_(0→*T*), which is different from the one in refs 46, 60, and can no longer be interpreted as the local generator of parametric translation of *U*_*g*_(0→*T*) with respect to *g*. But the maximum of the Fisher information 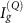 is still the squared gap between the maximum and minimum eigenvalues of *h*_*g*_(*T*), as in the case of static Hamiltonians^62^. Therefore, the key to determining the optimal estimation precision for the parameter *g* is finding *h*_*g*_(*T*) and its maximum and minimum eigenvalues.

Usually the evolution under a time-dependent Hamiltonian *H*_*g*_(*T*) is represented by the time-ordered exponential of *H*_*g*_(*T*), but it is complex and not convenient for our problem. Here we take an alternative approach that breaks the unitary evolution *U*_*g*_(0→*T*) into products of small time intervals Δ*t* and takes the limit Δ*t*→0. Interestingly, it turns out that with this approach, the maximum Fisher information (and the optimal quantum control) can be obtained without knowing the exact solution to *U*_*g*_(0→*T*). We show in Supplementary Note 1 that such an approach leads to


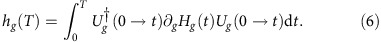


Obviously, it still includes the unitary evolution *U*_*g*_(0→*t*), which is unknown. However, it has the advantage that it is an integral over the time *t*, which makes it possible to decompose the global optimization of the eigenvalues of *h*_*g*_(*T*) into local optimizations at each time point *t*. The idea is that, as is known, the maximum eigenvalue of an Hermitian operator must be its largest expectation value over all normalized states, so the maximum eigenvalue of *h*_*g*_(*T*) is the maximum time integral of 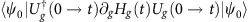 from 0 to *T* over all 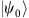. Considering *U*_*g*_(0→*T*) is unitary, *U*_*g*_(0→*t*)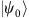 is also a normalized state, so the upper bound of 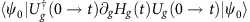 must be the maximum eigenvalue of *δ*_*g*_*H*_*g*_(*t*) at time *t*, which can be denoted as *μ*_max_(*t*). From this, it can be immediately inferred that the maximum eigenvalue of *h*_*g*_(*T*) is upper bounded by 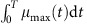. Similarly, the minimum eigenvalue of *h*_*g*_(*T*) is lower bounded by 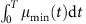. With these two bounds for the maximum and minimum eigenvalues of *h*_*g*_(*T*), respectively, we finally arrive at the upper bound of the quantum Fisher information 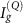,


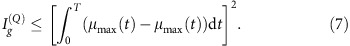


It shows that the upper bound of the quantum Fisher information 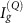 is determined by the integral of the gap between the maximum and minimum eigenvalues of *δ*_*g*_*H*_*g*_(*t*) from time 0 to *T*. It can straightforwardly recover the quantum Fisher information for a time-independent Hamiltonian *H*_*g*_ by identifying *μ*_max_(*t*) at all times *t* and identifying *μ*_min_(*t*) at all times *t*, respectively. And when *H*_*g*_=*gH*_0_, the maximum Fisher information is just *T*^2^Δ^2^, where Δ is the gap between the maximum and minimum eigenvalues of *H*_0_, the same as the result in ref. 62.

### Optimal Hamiltonian control

A question that naturally arises from the above result is whether the upper bound of quantum Fisher information 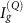 (7) is achievable. From the above derivation of the upper bound of 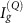, it is obvious that the upper bound cannot be saturated generally, unless there exists initial states 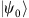 and 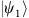 of the system such that *U*_*g*_(0→*t*)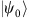 and *U*_*g*_(0→*t*)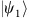 are the instantaneous eigenstates of *δ*_*g*_*H*_*g*_(*t*) with the maximum and minimum eigenvalues, respectively, at any time *t*. This imposes two conditions: (i) there exist 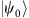 and 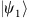, which are the eigenstates of 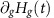 with the maximum and minimum eigenvalues at the initial time *t*=0; (ii) *U*_*g*_(0→*t*)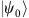 and *U*_*g*_(0→*t*)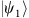 should remain as the eigenstates of *δ*_*g*_*H*_*g*_(*t*) with the maximum and minimum eigenvalues for all *t* under the evolution of *H*_*g*_(*t*). The first condition is easy to satisfy, but the second one is difficult, since the time change of an instantaneous eigenstate of *δ*_*g*_*H*_*g*_(*t*) is generally different from the evolution under the Hamiltonian *H*_*g*_(*t*) when *H*_*g*_(*t*) does not commute with *δ*_*g*_*H*_*g*_(*t*) or *H*_*g*_(*t*) does not commute between different time points. This condition is the main obstacle to the saturation of the upper bound of Fisher information (7).

However, it inspires us to think that if we can add some control Hamiltonian, which is independent of the parameter *g*, to the original Hamiltonian, so that the state evolution under the total Hamiltonian is the same as the time change of the instantaneous eigenstates of *δ*_*g*_*H*_*g*_(*t*), then a state starting from the eigenstate of *δ*_*g*_*H*_*g*_(0) with the maximum or minimum eigenvalue will always stay in that eigenstate of *δ*_*g*_*H*_*g*_(*t*) at any time *t*. And the upper bound of quantum Fisher information 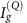 can then be achieved by preparing the system in an equal superposition of the eigenstates of *δ*_*g*_*H*_*g*_(*t*) with the maximum and minimum eigenvalues at the initial time *t*=0. So the key is finding such a control Hamiltonian.

A convenient way to realize the above target is to let each eigenstate of *δ*_*g*_*H*_*g*_(*t*) stay in the same eigenstate of *δ*_*g*_*H*_*g*_(*t*) at all times *t* when evolving under the total Hamiltonian. (Actually *δ*_*g*_*H*_*g*_(*t*) should be replaced by the derivative of total Hamiltonian now, but they are the same because the control Hamiltonian must be independent of *g*.) It implies that the *k*th eigenstate 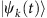 of *δ*_*g*_*H*_*g*_(*t*) should satisfy the Schrödinger equation 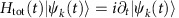, where *H*_tot_(*t*) denotes the total Hamiltonian. Unlike the usual situations where we know the Hamiltonian and want to find the solution to the state, here we know the solution to the state, 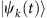, and need to find the appropriate Hamiltonian *H*_tot_(*t*) that directs the evolution instead. A simple solution to this equation is 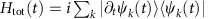. (Note this solution is Hermitian because 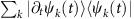 is skew-Hermitian.) Considering every eigenstate 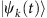 satisfies the *U*(1) symmetry, that is, multiplying 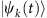 by an arbitrary phase 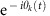 does not change that state, *H*_tot_(*t*) can be generalized to include an additional term 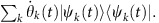 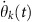
can be replaced by arbitrary real functions *f*_*k*_(*t*), and 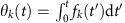. Thus, the optimal control Hamiltonian *H*_c_(*t*) finally turns out to be


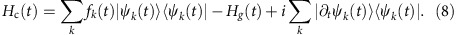


It will be seen in the examples below that proper choices of the functions *f*_*k*_(*t*) can significantly simplify the control Hamiltonian *H*_c_(*t*) in some cases.

**Figure 2:**
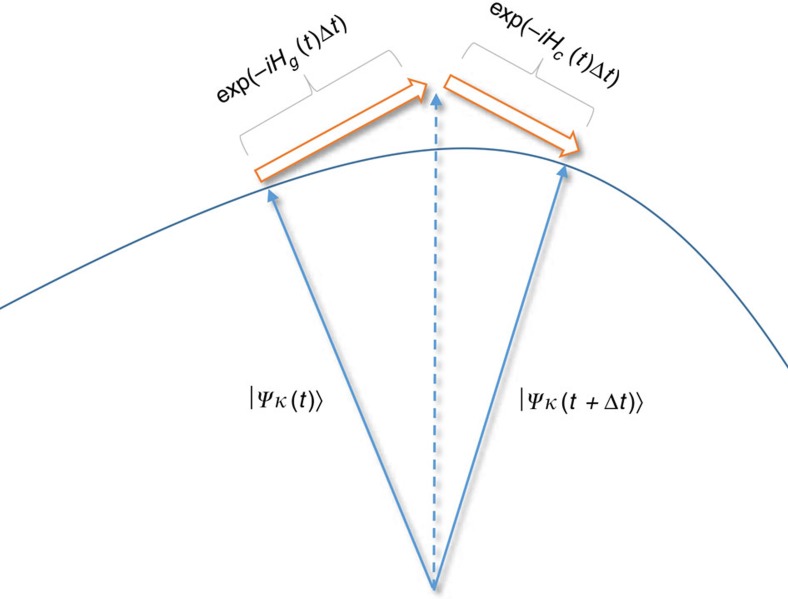
Optimal Hamiltonian control scheme. To achieve the maximum Fisher information, the optimal control Hamiltonian needs to keep the eigenstates of *δ*_*g*_*H*_*g*_(*t*) evolving along the tracks of the eigenstates of *δ*_*g*_*H*_*g*_(*t*) under the total Hamiltonian. The evolution under the total Hamiltonian *H*_*g*_(*t*)+*H*_c_(*t*) for a short time Δ*t* can be approximated as exp(−*iH*_c_(*t*)Δ*t*)exp(−*iH*_*g*_(*t*)Δ*t*). When exp(−*iH*_*g*_(*t*)Δ*t*) is applied on an eigenstate 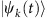 of *δ*_*g*_*H*_*g*_(*t*), the resulted state (represented by the dashed ray in the figure) is not necessarily still the instantaneous eigenstate 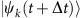 at time *t*+Δ*t*, and the role of the control Hamiltonian *H*_c_(*t*) is to pull the state back to the instantaneous eigenstate 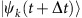 at time *t*+Δ*t*. In this way, with the assistance of the control Hamiltonian *H*_c_(*t*), each eigenstate 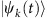 of *δ*_*g*_*H*_*g*_(*t*) will always evolve along the track of that eigenstate at any time *t*.

The role of this control Hamiltonian is to steer the eigenstates of *δ*_*g*_*H*_*g*_(*t*) evolving along the ‘tracks’ of the eigenstates of *δ*_*g*_*H*_*g*_(*t*) under the total Hamiltonian, which is the path to gain the most information about *g*, instead of being deviated off the ‘tracks’ by the original Hamiltonian *H*_*g*_(*t*). This is critical to the saturation of the upper bound of Fisher information. A schematic sketch for the role of the optimal control Hamiltonian *H*_c_(*t*) is plotted in Fig. 2. It is worth mentioning that similar ideas have been pursued in other works^63^,^64^,^65^,^66^ to steer the states of quantum systems along certain paths, such as the instantaneous eigenstates of Hamiltonians, with proper control fields.

It can be straightforwardly verified that with the above control Hamiltonian, the eigenstates 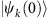 of *δ*_*g*_*H*_*g*_(*t*) at *t*=0 are the eigenstates of *h*_*g*_(*t*) for any time *t*, and the corresponding eigenvalues are 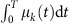, where *μ*_*k*_(*t*) is the *k*th eigenvalue of *δ*_*g*_*H*_*g*_(*t*) at time *t*. Therefore, *H*_c_(*t*) indeed gives the demanded control on the Hamiltonian to reach the upper and lower bounds of the eigenvalues of *h*_*g*_(*t*), and the upper bound of the quantum Fisher information 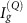 (7) can then be achieved by simply preparing the system in an equal superposition of the eigenstates of *δ*_*g*_*H*_*g*_(*t*) with the maximum and minimum eigenvalues at the initial time *t*=0 and making proper measurements on the system after an evolution of time *T*. The optimal measurement that gains the maximum Fisher information is generally a projective measurement along the basis 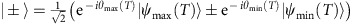, where 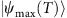 and 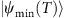 are the eigenstates of *δ*_*g*_*H*_*g*_(*t*) with the maximum and minimum eigenvalues at time *t*=*T*, and *θ*_max_(*T*) and *θ*_min_(*T*) are the additional phases of 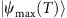 and 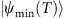 depending on the choice of *f*_*k*_(*t*) in the optimal control Hamiltonian (8). The details of the measurement scheme are discussed in Supplementary Note 2.

It is worth noting that the optimal control Hamiltonian (8) involves the estimated parameter *g*. However, *g* is unknown, so it should be replaced with a known estimate of *g*, say *g*_c_, in practice, and the control Hamiltonian becomes


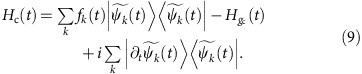


where *g* has been replaced by *g*_c_ and 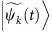 denotes the *k*th eigenstate of *δ*_*g*_*H*_*g*_(*t*) with *g*=*g*_c_.

The estimate *g*_c_ can be first obtained by some estimation scheme without the Hamiltonian control, then applied in the control Hamiltonian to have a more precise estimate of *g*. The new estimate of *g* can be fed back to the control Hamiltonian to further update the estimate of *g*. Thus, the above Hamiltonian control scheme is essentially adaptive, requiring feedback from each round of estimation to refine the control Hamiltonian and optimize the estimation precision.

We stress that the control Hamiltonian (9) is independent of the parameter *g*, although the optimal control Hamiltonian (8) involves *g*, otherwise the control Hamiltonian would carry additional information about *g*, which is not physical. From a quantum state discrimination point of view, the estimation of *g* is essentially to distinguish between 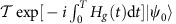 and 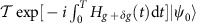, where 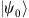 is the initial state of the system. When a control Hamiltonian *H*_c_(*t*, *g*_c_) is applied (where *g*_c_ is explicitly denoted), the two states become 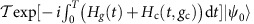 and 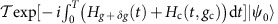. One can see that when *g* has a virtual shift *δg* in the original Hamiltonian, *g*_c_ is unchanged in the control Hamiltonian. The parameter *g*_c_ in the control Hamiltonian is always a constant (even when it is equal to the real value of *g*), while the parameter *g* in the original Hamiltonian is a variable. This is how the control Hamiltonian is independent of *g*. The appearance of *g* in the optimal control Hamiltonian (8) just indicates what *g*_c_ maximizes the Fisher information, and it turns out to be the real value of *g*.

As a simple verification of the above results, we show how the current results can recover the known ones in quantum metrology with time-independent Hamiltonians. Consider estimating a multiplicative parameter *g* in a time-independent Hamiltonian *H*_*g*_=*gH*_0_, which is the simplest case that has been widely studied. In this case, *δ*_*g*_*H*_*g*_(*t*)=*H*_0_ and 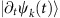
=0. To obtain a simple control Hamiltonian, we can choose *f*_*k*_(*t*) to be the *k*th eigenvalue *E*_*k*_ of *H*_0_, that is, multiply 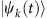 with a phase 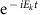, in the optimal control Hamiltonian *H*_c_(*t*) (8); then *H*_c_(*t*)=0. This implies no Hamiltonian control is necessary for this case, in accordance with the result in ref. 62.

A more general case is that the Hamiltonian is still independent of time but the parameter to estimate is not necessarily multiplicative. This has attracted a lot of attention recently^46^,^47^,^48^,^50^,^51^,^52^,^67^. The Hamiltonian in this case can be represented as *H*_*g*_(*t*)=*H*_*g*_ in general. Since the Hamiltonian is still time-independent, we have 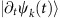
=0. So, the optimal control Hamiltonian is 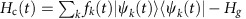. But in this case, 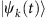 are not necessarily the eigenstates of *H*_*g*_, and 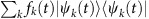 cannot cancel *H*_*g*_ generally. To simplify *H*_c_(*t*), we can simply choose *f*_*k*_(*t*)=0, then *H*_c_(*t*)=−*H*_*g*_. It implies a reverse of the original Hamiltonian can lead to the maximum Fisher information in this case. This recovers the result in ref. 52, which showed that the optimal control to maximize the quantum Fisher information for this case is just to apply a reverse of the original unitary evolution at each time point. Of course, this is not the unique solution to *H*_c_(*t*), and a large family of solutions exist corresponding to different choices of *f*_*k*_(*t*), all leading to the maximum Fisher information.

### Estimation of field amplitude

To exemplify the features of quantum metrology with time-dependent Hamiltonians and the power of the above Hamiltonian control scheme, we consider a simple physical example below. This example will show some important characteristics of time-dependent quantum metrology and how the optimized Hamiltonian control can dramatically boost the estimation precision.

Let us consider a qubit in a uniformly rotating magnetic field, 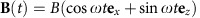, where **e**_*x*_ and **e**_*z*_ are the unit vectors in the 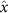 and 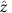 directions, respectively, and we want to estimate the amplitude *B* or the rotation frequency *ω* of the field. To acquire the information about the magnetic field, we let the qubit evolve in the field for some time *T*, then measure the final state of the qubit to learn *B* or *ω*. The interaction Hamiltonian −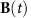
·***σ*** between the qubit and the field is


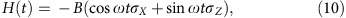


where we assumed the magnetic moment of the qubit to be 1.

We first consider estimating the amplitude *B* of the magnetic field. It is easy to verify that the derivative of *H*(*t*) with respect to *B* has eigenvalues ±1 for any *t*, therefore, the maximum quantum Fisher information (7) of estimating *B* at time *T* is


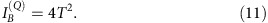


As shown previously, it requires some control on the Hamiltonian to reach this maximum quantum Fisher information. It can be straightforwardly obtained that the eigenstates of *δ*_*B*_*H*(*t*) are 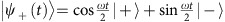 and 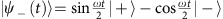, where 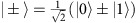, corresponding to oscillations in the *Z*−*X* plane. Since *δ*_*B*_*H*(*t*)=*B*^−1^*H*(*t*), we can choose the first term in equation (8) to cancel *H*(*t*). Then, the optimal control Hamiltonian *H*_c_(*t*) (8) is


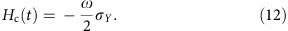


What about if we do not apply the control Hamiltonian *H*_c_(*t*)? We obtain the evolution of the qubit and the quantum Fisher information for the amplitude *B* without any Hamiltonian control in Supplementary Note 3. The quantum Fisher information for this case is


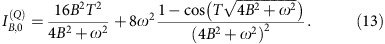


It implies that when 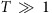,


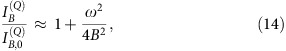


indicating that the increase of Fisher information by the Hamiltonian control is determined by the ratio between *ω* and *B*.

It is interesting to note that if the field rotation frequency *ω* is small, the increase in Fisher information by the Hamiltonian control would be small as well, as shown by equation (14). This is because when 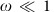, the magnetic field is changing so slowly that the evolution of the qubit state is approximately adiabatic, and an eigenstate of *δ*_*B*_*H*(*t*) would always stay in that eigenstate considering *δ*_*B*_*H*(*t*) commutes with *H*(*t*). Thus, the condition for optimizing the Fisher information can be automatically satisfied, and the maximum Fisher information is achieved as a result. This is also verified by equation (12) that when 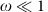, the optimal control Hamiltonian is close to zero, which means almost no Hamiltonian control is necessary for this case.

**Figure 3:**
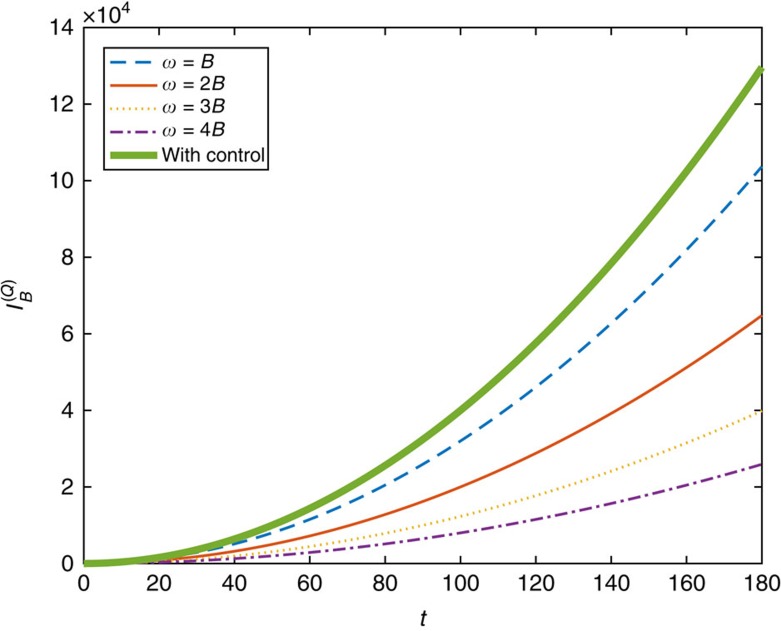
Quantum Fisher information for the field amplitude. Quantum Fisher information 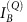 for the amplitude *B* of the rotating magnetic field **B**(*t*) versus the evolution time *t* is plotted for different choices of rotation frequency *ω* without the Hamiltonian control, and compared to that with the optimized Hamiltonian control. The true value of *B* in the figure is 1. It can be observed that when *ω* is large compared to the amplitude of the magnetic field *B*, the Fisher information becomes small. The Fisher information with the optimal Hamiltonian control is the highest, whatever *ω* is, which verifies the advantage of Hamiltonian control for this case.

The quantum Fisher information of *B* is plotted for different rotation frequencies *ω* without the control Hamiltonian and compared to that with the optimal control Hamiltonian (12) in Fig. 3.

### Estimation of field rotation frequency

Now, we turn to the estimation of the rotation frequency *ω* of the magnetic field. Frequency measurement is important in many areas of physics, and has been widely studied in different contexts, for example, a single-spin spectrum analyzer^68^. High-precision phase estimation has been realized in many experiments in recent years, for example, on a single nuclear spin in diamond with a precision of order *T*^−0.85^ by Waldherr *et al*.^69^

To study the estimation precision of the frequency *ω*, note *δ*_*ω*_*H*(*t*) is *tB*(sin *ωtσ*_*X*_−cos *ωtσ*_*Z*_). The eigenvalues of *δ*_*ω*_*H*(*t*) are *μ*(*t*)=±*tB* then, so the maximum and minimum eigenvalues of are 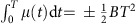. Therefore, the maximum Fisher information of estimating *ω* is


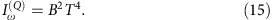


The eigenstates of *δ*_*ω*_*H*(*t*) are 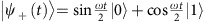 and 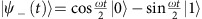. If we choose *f*_*k*_(*t*)=0 for equation (8), then the optimal control Hamiltonian is


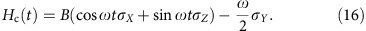


The first term in *H*_c_(*t*) (16) cancels the original Hamiltonian *H*(*t*), so that the eigenstates of *δ*_*ω*_*H*(*t*) would not be deviated by *H*(*t*), and the second term in *H*_c_(*t*) guides the eigenstates of *δ*_*ω*_*H*(*t*) along the tracks of those eigenstates during the whole evolution under the total Hamiltonian with the control *H*_c_(*t*).

The above result of 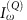 has an important implication: it is known that the time scaling of Fisher information for a parameter of a time-independent Hamiltonian is at most *T*^2^, even with some control on the Hamiltonian, a fundamental limit in time-independent quantum metrology^52^; however, in this example, the time scaling of Fisher information for the frequency *ω* reaches *T*^4^, an order *T*^2^ higher than the time-independent limit! This indicates that some fundamental limits in the time-independent quantum metrology no longer hold when the Hamiltonian becomes varying with time, and they can be dramatically violated in the presence of appropriate quantum control on the system, showing a significant discrepancy between the time-dependent and the time-independent quantum metrology.

An interesting question that naturally arises is if there is no control Hamiltonian *H*_c_(*t*), can the maximum Fisher information 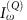 still scale as *T*^4^? In Supplementary Note 3, we derive the maximum Fisher information for the rotation frequency *ω* in the absence of Hamiltonian control by an exact computation of the qubit evolution in the rotating magnetic field, and the result turns out to be


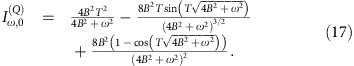


Therefore, without any Hamiltonian control on the system, the Fisher information would still scale as *T*^2^ as in time-independent quantum metrology, which is substantially lower than the *T*^4^ scaling with the optimized Hamiltonian control. This exhibits the advantage of Hamiltonian control in enhancing time-dependent quantum metrology.

**Figure 4:**
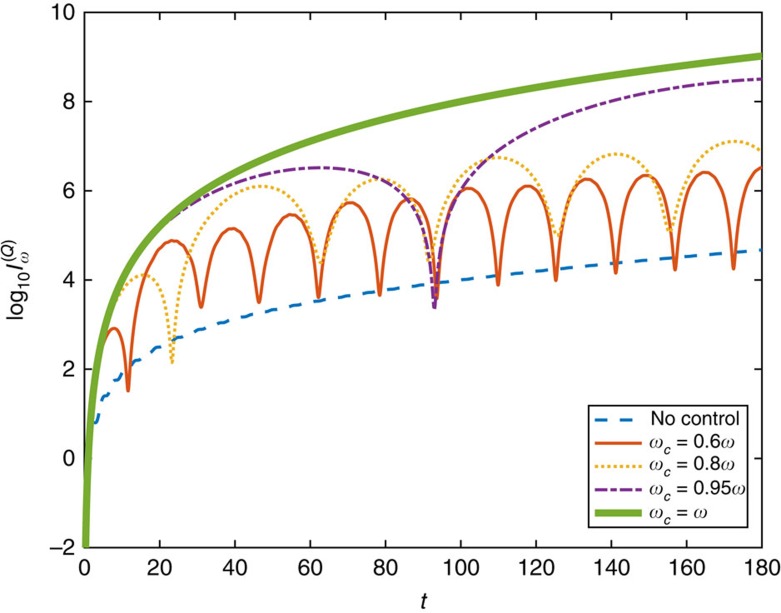
Quantum Fisher information for the field frequency. The logarithm (base 10) of the quantum Fisher information 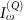 for the rotation frequency *ω* of the magnetic field **B**(*t*) versus the evolution time *t* is plotted for different trial values *ω*_c_ of the rotation frequency, and compared to the Fisher information in the absence of the control Hamiltonian *H*_c_(*t*). The real value of *B* and the real value of *ω* are both 1. It can be observed that, even with some sub-optimal choices of *ω*_c_ that is not equal to the real value of *ω*, the scaling of the Fisher information can still be much higher than that without any Hamiltonian control, and when *ω*_c_ approaches the real rotation frequency *ω* of the magnetic field, higher Fisher information can be gained with the assistance of Hamiltonian control. When *ω*_c_=*ω*, the Fisher information reaches the maximum, which confirms the theoretical results.

Figure 4 plots the Fisher information of *ω* in the presence of the control Hamiltonian with various *ω*_c_, and compares it to that without the control Hamiltonian.

It should be noted that when the Hamiltonian is allowed to vary with time, the time scaling of Fisher information may be raised in a trivial way: the strength or the level gap of the Hamiltonian may itself increase rapidly with time. For example, if the Hamiltonian is growing exponentially with time (for example, *H*_*g*_=*ge*^*t*^*σ*_*Z*_), the Fisher information can have an exponential time scaling. The nontriviality of the current result lies in that the Hamiltonian (10) has a fixed gap 2*B* between its highest and lowest levels, which does not scale up with time, and thus the increase in Fisher information does not result from any time growth of the Hamiltonian.

One may be wondering about the origin of the *T*^4^ scaling. It is not from the control Hamiltonian, since the control Hamiltonian is independent of the estimated parameter, which is *ω* in this example. The *T*^4^ scaling originates from the dynamics of the original Hamiltonian. Consider two original Hamiltonians with slightly deviated parameters *ω* and *ω*+*δω*. The discrepancy between them is amplified by a time factor *t* as they evolve, and correspondingly the distance between the states evolving under these two Hamiltonians is amplified by a time factor *t* as well. Since the squared distance between two states with neighbouring parameters is approximately proportional to the quantum Fisher information as manifested by the Bures metric^61^, the quantum Fisher information of *ω* can therefore be increased by an order *T*^2^ after an evolution of time *T*. The control Hamiltonian helps keep the qubit on the optimal route that gains the most Fisher information.

### Adaptive control for frequency estimation

A notable point in the above Hamiltonian control scheme for frequency estimation is that the optimal control Hamiltonian *H*_c_(*t*) (16) involves the rotation frequency *ω*. However, *ω* is the parameter to estimate, so, in practice, we can only use an estimate of *ω*, say *ω*_c_, instead of the real value of *ω* in implementing the control Hamiltonian (16), and the control Hamiltonian would actually be


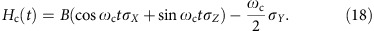


When the measurement runs for multiple rounds, the estimate *ω*_c_ will approach the real value of *ω*, and the optimal Fisher information (15) can be saturated by adaptively updating the estimate of *ω* in the control Hamiltonian. This implies that a feedback of the information about *ω* from each round of measurement into the next round is necessary to implement the optimal Hamiltonian control scheme and maximize the estimation precision for *ω*.

The details of the adaptive Hamiltonian control scheme are presented in Supplementary Note 5. Generally one needs to first obtain an initial estimate of *ω* by some estimation scheme without the Hamiltonian control, then apply it to the control Hamiltonian and update it by estimation in the presence of the control Hamiltonian. The updated estimate of *ω* can again be applied in the control Hamiltonian to produce a better estimate of *ω*, and so forth.

An important point shown in Supplementary Note 4 is that with an estimate of *ω*, *ω*_c_, which deviates from the exact value of *ω* by *δω*, *δω*=*ω*_c_−*ω*, the Fisher information in the presence of the Hamiltonian control is approximately


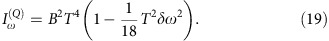


So, to approach the *T*^4^ scaling of Fisher information for a given evolution time *T*, the necessary precision *δω* of the estimate *ω*_c_ in the control Hamiltonian is only of the order *T*^−1^, so the feedback of a low-precision estimate of *ω* in the Hamiltonian control can lead to a high-precision estimate of *ω*. This lays the foundation for the adaptive Hamiltonian control scheme. In particular, it implies that the precision of the initial estimate of *ω* also just needs to be of the order *T*^−1^, attainable in the absence of Hamiltonian control, which is exactly what we need.

In fact, such an iterative feedback control scheme can approach the *T*^4^ scaling of Fisher information very efficiently. It is shown in Supplementary Note 5 that the number of necessary rounds of feedback control to realize the *T*^4^ scaling for a large *T* is only


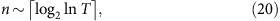


a double logarithm of *T*, so very few rounds of feedback control are necessary to approach the *T*^4^ scaling.

It is also worth mentioning that there is a minimum precision requirement of the initial estimation of *ω* without the Hamiltonian control, so that the Fisher information increases after each round of feedback control:


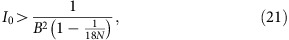


where *N* is the number of measurements in each round of feedback control, otherwise the Fisher information would decrease as the feedback control proceeds.

## Discussion

The final problem we want to discuss about the above optimal Hamiltonian control scheme for time-dependent quantum metrology is the case that the eigenstate of *δ*_*g*_*H*_*g*_(*t*) with the maximum or minimum eigenvalue does not always stay in the same eigenstate during the evolution. In deriving the optimal control Hamiltonian (8), we let each eigenstate of *δ*_*g*_*H*_*g*_(*t*) stay in the same eigenstate during the evolution for simplicity. This implicitly assumes that the eigenstate of *δ*_*g*_*H*_*g*_(*t*) with the maximum or minimum eigenvalue also stays in the same eigenstate during the evolution. However, if the highest or lowest level crosses other levels of *δ*_*g*_*H*_*g*_(*t*), the corresponding eigenstate will change from one eigenstate of *δ*_*g*_*H*_*g*_(*t*) to another at the crossing.

In the presence of such a level crossing, the upper bound of the maximum eigenvalue of *h*_*g*_(*T*) or the lower bound of the minimum eigenvalue of *h*_*g*_(*T*) cannot be attained, and as a result the upper bound (7) on the quantum Fisher information cannot be saturated. In particular, if the highest and lowest levels of *δ*_*g*_*H*_*g*_(*t*) cross each other, the Fisher information will even drop after the crossing, because the gap between the maximum and minimum eigenvalues of *h*_*g*_(*T*) will shrink. Thus, it is necessary to cancel or suppress the effect of level crossing in *δ*_*g*_*H*_*g*_(*t*) to maximize the Fisher information.

To keep the highest or lowest level of *δ*_*g*_*H*_*g*_(*t*) still in the highest or lowest level after a crossing in *δ*_*g*_*H*_*g*_(*t*), we need to change the dynamics of the system near the crossing so that the highest or lowest level of *δ*_*g*_*H*_*g*_(*t*) before the level crossing transits to the new one after the level crossing. We propose an additional Hamiltonian control scheme in the Methods to realize such a transition.

**Figure 5:**
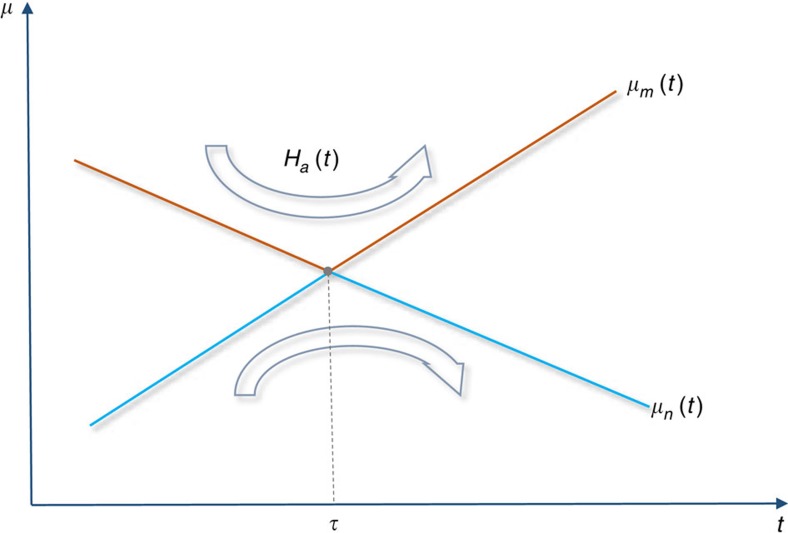
Additional Hamiltonian control scheme at level crossings of *δ*_*g*_*H*_*g*_(*t*). Additional Hamiltonian control is necessary to eliminate the effect of a crossing between the highest/lowest level and another level of *δ*_*g*_*H*_*g*_(*t*). The role of the additional control Hamiltonian *H*_a_(*t*) is to transform the original instantaneous highest/lowest level to the new instantaneous highest/lowest level of *δ*_*g*_*H*_*g*_(*t*). Suppose the red curve in the figure is the highest level of *δ*_*g*_*H*_*g*_(*t*). Before 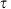, 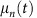 is the highest level of *δ*_*g*_*H*_*g*_(*t*). At time 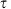, 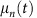 crosses the level 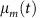, which becomes the highest level after the crossing. The additional control Hamiltonian *H*_a_(*t*) is to transit the highest level from 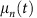 to 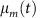 at time 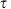. The argument is similar if the blue curve is the lowest level of *δ*_*g*_*H*_*g*_(*t*).

In Fig. 5, the role of the additional Hamiltonian control is plotted. When there are multiple crossings between the highest/lowest level and other levels of *δ*_*g*_*H*_*g*_(*t*) during the whole evolution process, there must be an additional Hamiltonian control applied at each level crossing.

## Methods

### Additional quantum control at level crossings of ***δ***_*g*_ *H*_*g*_(*t*)

Suppose a crossing occurs between the highest or lowest level and another level of *δ*_*g*_*H*_*g*_(*t*) at time 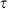. 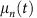 is the highest or lowest level of *δ*_*g*_*H*_*g*_(*t*) before 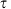 while 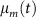 becomes the highest or lowest level after 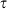, and 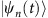 and 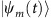 are the corresponding eigenstates. Intuitively, the following *σ*_*X*_-like control Hamiltonian


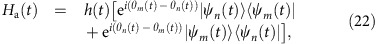


should rotate 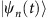 to 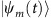, with *h*(*t*) to be some time-dependent control parameters and 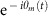, 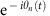 to be the additional phases of 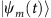, 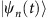 determined by the choices of *f*_*m*_(*t*), *f*_*n*_(*t*) in the optimal control Hamiltonian (8). In order not to affect the additional Hamiltonian controls at other level crossings, *H*_a_(*t*) must be completed within a sufficiently short time *δt*. As shown in Supplementary Note 6, the control parameter *h*(*t*) must satisfy


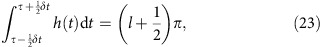


where *l* is an arbitrary integer, so that the system can be exactly transferred to the new eigenstate 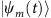 from 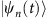 by the additional control Hamiltonian.

An intuitive idea why the above additional control Hamiltonian *H*_a_(*t*) can drive 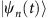 to 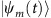 can be understood as follows. Note that the total Hamiltonian is the sum of *H*_*g*_(*t*), *H*_c_(*t*) and the additional control Hamiltonian *H*_a_(*t*) now. According to the time-dependent generalization of the Suzuki–Trotter product formula^70^, if we break the time interval 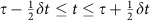 into many small pieces at properly sampled time points *t*_1_, *t*_2_, ⋯, *t*_*n*_, the total evolution of the system from 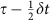 to 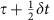 can be approximated as the time-ordered product of 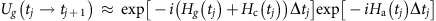, where Δ*t*_*j*_=*t*_*j*+1_−*t*_*j*_, implying that at each short time piece Δ*t*_*j*_, the state 
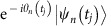
 is slightly shifted to 
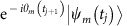
 by *H*_a_(*t*_*j*_), following which 
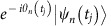
 is shifted to 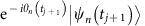 and 
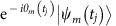
 is shifted to 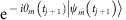 by *H*_*g*_(*t*_*j*_)+*H*_c_(*t*_*j*_). Thus, the total effect of the additional control Hamiltonian *H*_a_(*t*), along with the original Hamiltonian *H*_*g*_(*t*) and the control Hamiltonian *H*_c_(*t*), is continuously driving the system from 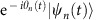 to 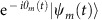, where 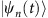 and 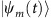 are also changing at the same time.

A rigorous analysis for the additional Hamiltonian *H*_a_(*t*) is given in Supplementary Note 6. It turns out that in a rotating frame where all 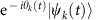 are static, the total Hamiltonian is transformed to *H*′(*t*)=*h*(*t*)*σ*_*mn*_, where *σ*_*mn*_ is a *σ*_*X*_-like transition operator between two static basis states 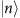 and 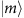 in the new frame that correspond to 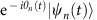 and 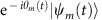 in the original frame. This indicates that in the presence of the additional control Hamiltonian *H*_a_(*t*), 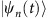 can be transited to 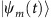 continuously around the level crossing between 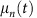 and 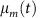.

It should be noted that an additional phase 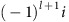 will be introduced to the eigenstates 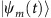 and 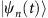 of *δ*_*g*_*H*_*g*_(*t*) by the additional Hamiltonian control. This may change the relative phase of the system when it is in a superposed state involving 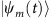 or 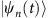 and needs to be taken into account in that case. The detail about the additional phase is given in Supplementary Note 6.

### Data availability

The code and data used in this work are available on request to the corresponding author.

## Additional information

**How to cite this article:** Pang, S. & Jordan, A.N. Optimal adaptive control for quantum metrology with time-dependent Hamiltonians. *Nat. Commun.* **8,** 14695 doi: 10.1038/ncomms14695 (2017).

**Publisher's note**: Springer Nature remains neutral with regard to jurisdictional claims in published maps and institutional affiliations.

## Supplementary Material

Supplementary Information Supplementary Notes and Supplementary References.
