# Supplementary material for: Optimal adaptive control for quantum metrology with time-dependent Hamiltonians
Source: Nat Commun. 2017 Mar 9;8:14695. doi: 10.1038/ncomms14695 (PMC5512879; doi:10.1038/ncomms14695)
Supplement: Supplementary Information — Supplementary Notes and Supplementary References. [file ncomms14695-s1.pdf]

SUPPLEMENTARY NOTE 1. DERIVATION OF $h_g(T)$

In this Supplementary Note, we derive $h_g(T)$ for a time-dependent Hamiltonian $H_g(t)$, defined as

$$h_g(T) = iU_g^\dagger(0 \to T)\partial_g U_g(0 \to T), \tag{1}$$

where $U_g(0 \to T)$ is the unitary evolution under the time-dependent Hamiltonian $H_g(t)$, and it determines the quantum Fisher information of estimating g in the following way,

$$I_g^{(Q)} = 4\langle\psi_0|\Delta^2 h_g(T)|\psi_0\rangle, \tag{2}$$

where $|\psi_0\rangle$ is the initial state of the system. The maximum quantum Fisher information is the square of the gap between the maximum and minimum eigenvalues of $h_g(T)$.

In order to obtain $h_g(T)$, we break the unitary evolution $U_g(0 \to T)$ for a time duration T into small time intervals Δt,

$$U_g(0 \to T) = U_g(T-\Delta t \to T)U_g(T-2\Delta t \to T-\Delta t)\cdots U_g(\Delta t \to 2\Delta t)U_g(0 \to \Delta t). \tag{3}$$

Then,

$$\begin{aligned}\partial_g U_g(0 \to T) = \sum_{k=0}^{T/\Delta t-1} &\Big\{U_g(T-\Delta t \to T)\cdots U_g((k+1)\Delta t \to (k+2)\Delta t) \\ &[\partial_g U_g(k\Delta t \to (k+1)\Delta t)]U_g((k-1)\Delta t \to k\Delta t)\cdots U_g(0 \to \Delta t)\Big\}.\end{aligned} \tag{4}$$

When the time interval Δt is sufficiently small, the Hamiltonian $H_g(t)$ is approximately time-independent during each time interval $k\Delta t \le t \le (k+1)\Delta t$, i.e.,

$$U_g(k\Delta t \to (k+1)\Delta t) \approx \exp(-iH_g(k\Delta t)\Delta t), \tag{5}$$

and by expanding $\exp(-iH_g(k\Delta t)\Delta t)$ to the first order of Δt, we have

$$\exp(-iH_g(k\Delta t)\Delta t) = I - i\Delta t H_g(k\Delta t) + O(\Delta t^2), \tag{6}$$

so,

$$\partial_g U_g(k\Delta t \to (k+1)\Delta t) \approx -i\Delta t\partial_g H_g(k\Delta t) + O(\Delta t^2). \tag{7}$$

In the limit $\Delta t \to 0$, $\partial_g U_g(0 \to T)$ can be written in the following integral form,

$$\partial_g U_g(0 \to T) = -i\int_0^T U_g(t \to T)\partial_g H_g(t)U_g(0 \to t)\mathrm{d}t, \tag{8}$$

which is the exact solution to $\partial_g U_g(0 \to T)$.

When $U_g^\dagger(0 \to T)$ is multiplied to $\partial_g U_g(0 \to T)$ from the left, since

$$U_g^\dagger(0 \to T)U_g(t \to T) = U_g^\dagger(0 \to t), \tag{9}$$

$h_g(T)$ in Eq. (1) turns out to be

$$h_g(T) = \int_0^T U_g^\dagger(0 \to t)\partial_g H_g(t)U_g(0 \to t)\mathrm{d}t. \tag{10}$$

This gives the integral form of $h_g(T)$ in the main text for a time-dependent Hamiltonian $H_g(t)$ at time $t = T$. When $H_g(t)$ is independent of time, Eq. (10) degenerates to the relevant formula in [1].

SUPPLEMENTARY NOTE 2. MEASUREMENT SCHEME FOR ESTIMATION WITH OPTIMAL HAMILTONIAN CONTROL

In this Supplementary Note, we derive the estimator and the measurement scheme that can gain the upper bound of Fisher information given by Eq. (7) in the main manuscript with the assistance of the optimal Hamiltonian control.

Suppose the parameter that we want to estimate is g. According to Eq. (8) of the main manuscript, the optimal control Hamiltonian is

$$H_{\rm c}(t) = \sum_k f_k(t)|\psi_k(t)\rangle\langle\psi_k(t)| - H_g(t) + i\sum_k |\partial_t\psi_k(t)\rangle\langle\psi_k(t)|, \tag{11}$$

where $H_g(t)$ is the original Hamiltonian with the parameter g, $|\psi_k(t)\rangle$ are the eigenstates of $\partial_g H_g(t)$ and $f_k(t)$ are arbitrary real functions. Since we do not know the exact value of g, the parameter g in the optimal control Hamiltonian (11) should be replaced by some known estimate of g, $g_{\rm c}$, in practice, and the control Hamiltonian is actually

$$H_{\rm c}(t) = \sum_k f_k(t)|\widetilde{\psi_k}(t)\rangle\langle\widetilde{\psi_k}(t)| - H_{g_{\rm c}}(t) + i\sum_k |\partial_t\widetilde{\psi_k}(t)\rangle\langle\widetilde{\psi_k}(t)|, \tag{12}$$

where $|\widetilde{\psi_k}(t)\rangle$ are also dependent on $g_{\rm c}$ instead of g, i.e., $|\widetilde{\psi_k}(t)\rangle$ are eigenstates of $\partial_g H_g(t)|_{g=g_{\rm c}}$.

It should be noted that the parameter $g_{\rm c}$ in the control Hamiltonian (12) is always a constant, even when it is equal to the real value of g, while the parameter g in the original Hamiltonian $H_g(t)$ is a variable. This should be kept in mind in computing the Fisher information for g. The appearance of g in the optimal control Hamiltonian (11) just indicates what value of $g_{\rm c}$ maximizes the Fisher information, and it turns out to be the real value of g.

When $g_{\rm c}$ is close to g, the total Hamiltonian can be written as

$$\begin{aligned} H_{\rm tot}(t) =& H_g(t) + H_{\rm c}(t) \\ =& \sum_k f_k(t)|\widetilde{\psi_k}(t)\rangle\langle\widetilde{\psi_k}(t)| + \partial_g H_g(t)|_{g=g_{\rm c}}\delta g + i\sum_k |\partial_t\widetilde{\psi_k}(t)\rangle\langle\widetilde{\psi_k}(t)|, \end{aligned} \tag{13}$$

up to the first order of δg, where $\delta g = g - g_{\rm c}$.

The evolution under $H_{\rm tot}(t)$ can be decomposed as

$$U(0\to T) = \lim_{\Delta t\to 0} \exp(-iH_{\rm tot}(T)\Delta t)\exp(-iH_{\rm tot}(T-\Delta t)\Delta t)\cdots\exp(-iH_{\rm tot}(0)\Delta t), \tag{14}$$

and at time t,

$$\exp(-iH_{\rm tot}(t)\Delta t) \approx \exp(-i\sum_k |\partial_t\widetilde{\psi_k}(t)\rangle\langle\widetilde{\psi_k}(t)|\Delta t)\exp(-i\sum_k f_k(t)|\widetilde{\psi_k}(t)\rangle\langle\widetilde{\psi_k}(t)|\Delta t)\exp(-i\partial_g H_g(t)|_{g=g_{\rm c}}\delta g\Delta t). \tag{15}$$

Note that generally different orderings of the three terms at the right side of Eq. (15) will give different results when Δt is finite, but when $\Delta t \to 0$, they will give the same result. The ordering chosen in (15) is for convenience of computation below.

If a system is initially in an eigenstate of $\partial_g H_g(t)|_{g=g_{\rm c}}$ at $t=0$, say $|\widetilde{\psi_k}(0)\rangle$, then according to Eq. (14) and (15), the state after an evolution of time T is

$$U(0\to T)|\widetilde{\psi_k}(0)\rangle = \exp\Big[-i\Big(\theta_k(T) + \delta g\int_0^T \mu_k(t){\rm d}t\Big)\Big]|\widetilde{\psi_k}(T)\rangle, \tag{16}$$

where

$$\theta_k(T) = \int_0^T f_k(t){\rm d}t. \tag{17}$$

Now, suppose the maximum and minimum eigenvalues of $\partial_g H_g(t)|_{g=g_{\rm c}}$ at time t are $\mu_{\max}(t)$ and $\mu_{\min}(t)$, and the corresponding eigenstates are $|\widetilde{\psi_{\max}}(t)\rangle$ and $|\widetilde{\psi_{\min}}(t)\rangle$, respectively. To achieve the maximum Fisher information given by Eq. (7) in the main manuscript, we can prepare the system in an equal superposition of $|\widetilde{\psi_{\max}}(0)\rangle$ and $|\widetilde{\psi_{\min}}(0)\rangle$ at the initial time $t=0$,

$$|\Psi(0)\rangle = \frac{1}{\sqrt{2}}(|\widetilde{\psi_{\max}}(0)\rangle + |\widetilde{\psi_{\min}}(0)\rangle). \tag{18}$$

Then after evolving for time T, the state of the system is

$$
\begin{aligned}
|\Psi(T)\rangle =&\frac{1}{\sqrt{2}}\Big\{\exp\Big[-i\Big(\theta_{\max}(T)+\delta g\int_0^T\mu_{\max}(t)\mathrm{d}t\Big)\Big]|\widetilde{\psi_{\max}}(T)\rangle\\
&+\exp\Big[-i\Big(\theta_{\min}(T)+\delta g\int_0^T\mu_{\min}(t)\mathrm{d}t\Big)\Big]|\widetilde{\psi_{\min}}(T)\rangle\Big\}.
\end{aligned}
\tag{19}
$$

Note that $\theta_{\max/\min}(t)$ denotes the value of $\theta_k(t)$ associated with the maximum or minimum eigenvalue of $\partial_g H_g(t)|_{g=g_c}$, but not the maximum or minimum over $\theta_k(t)$.

Since $\partial_g=\partial_{\delta g}$, the quantum Fisher information of estimating of g by measuring $|\Psi(T)\rangle$ is

$$
\begin{aligned}
I_g^{(Q)} =&4\Big(\langle\partial_{\delta g}\Psi(T)|\partial_{\delta g}\Psi(T)\rangle-|\langle\Psi(T)|\partial_{\delta g}\Psi(T)\rangle|^2\Big)\\
=&\Big[\int_0^T\big(\mu_{\max}(t)-\mu_{\min}(t)\big)\mathrm{d}t\Big]^2,
\end{aligned}
\tag{20}
$$

which is exactly the upper bound of quantum Fisher information given by Eq. (7) in the main manuscript.

To attain this quantum Fisher information, we can measure the following observable

$$
\mathcal{O}=|+\rangle\langle+|-|-\rangle\langle-|,
\tag{21}
$$

where

$$
|\pm\rangle=\frac{1}{\sqrt{2}}(e^{-i\theta_{\max}(T)}|\psi_{\max}(T)\rangle\pm e^{-i\theta_{\min}(T)}|\psi_{\min}(T)\rangle),
\tag{22}
$$

which are varying with time T. It is important to note that $|\pm\rangle$ are also dependent on g_c, instead of g, which makes it possible to implement the measurement of $\mathcal{O}$ without knowing the exact value of g.

It can be obtained that the expectation value and variance of $\mathcal{O}$ under the state $|\Psi(t)\rangle$ are

$$
\begin{aligned}
\langle\mathcal{O}\rangle =&\cos\Big[\int_0^T(\mu_{\max}(t)-\mu_{\min}(t))\delta g\mathrm{d}t\Big],\\
\langle\Delta\mathcal{O}^2\rangle =&\sin^2\Big[\int_0^T(\mu_{\max}(t)-\mu_{\min}(t))\delta g\mathrm{d}t\Big].
\end{aligned}
\tag{23}
$$

$\langle\mathcal{O}\rangle$ can be considered as an estimator of g (with some local unit difference characterized by $\partial_{\delta g}\langle\mathcal{O}\rangle$ and potential systematic errors which can be eliminated by calibration) since it is dependent on g. The parameter g can be obtained from $\langle\mathcal{O}\rangle$, and the variance of the estimate [2, 3] is

$$
\delta g^2=\frac{\langle\Delta\mathcal{O}^2\rangle}{|\partial_{\delta g}\langle\mathcal{O}\rangle|^2}=\frac{1}{\Big[\int_0^T(\mu_{\max}(t)-\mu_{\min}(t))\mathrm{d}t\Big]^2},
\tag{24}
$$

which exactly saturates the upper bound of Fisher information in Eq. (7) of the main manuscript. If there are N trials, the precision would be $1/\sqrt{N}$ of δg then.

SUPPLEMENTARY NOTE 3. FISHER INFORMATION FOR B AND ω IN THE ABSENCE OF HAMILTONIAN CONTROL

In this Supplementary Note, we derive the evolution of a qubit in a rotating magnetic field and the optimal quantum Fisher information for the amplitude B and the rotation frequency ω of the magnetic field in the absence of control Hamiltonian.

Suppose the rotating magnetic field is $\mathbf{B}(t)$,

$$
\mathbf{B}(t)=B(\cos\omega t\mathbf{e}_x+\sin\omega t\mathbf{e}_z),
\tag{25}
$$

where the amplitude of the field B is assumed to be constant for simplicity. The interaction Hamiltonian between a qubit and the field is

$$
H(t)=-\mathbf{B}(t)\cdot\boldsymbol{\sigma}=-B(\cos\omega t\sigma_X+\sin\omega t\sigma_Z).
\tag{26}
$$

When there is no control on the Hamiltonian, the evolution of the qubit is determined by the Schrödinger equation

$$i\partial_t|\Psi(t)\rangle = H(t)|\Psi(t)\rangle. \tag{27}$$

To derive the evolution of the qubit in this case, note that

$$\exp(i\frac{\omega}{2}\sigma_Y t)\sigma_X \exp(-i\frac{\omega}{2}\sigma_Y t) = \cos\omega t\sigma_X + \sin\omega t\sigma_Z. \tag{28}$$

We know that if a state under a general Hamiltonian $H(t)$ is transformed by $\exp(iH_0 t)$, the effective Hamiltonian for the evolution of the new state is

$$H'(t) = \exp(iH_0 t)H(t)\exp(-iH_0 t) - H_0. \tag{29}$$

Therefore, the rotating Hamiltonian (26) can be perceived as the effective Hamiltonian of an original Hamiltonian $-B\sigma_X + \frac{\omega}{2}\sigma_Y$ in a frame rotating as $\exp(i\frac{\omega}{2}\sigma_Y t)$.

The evolution of a qubit under the Hamiltonian $-B\sigma_X + \frac{\omega}{2}\sigma_Y$ is $\exp[i(B\sigma_X - \frac{\omega}{2}\sigma_Y)t]$, so the evolution under the rotating Hamiltonian (26) is

$$U(0\to t) = \exp(i\frac{\omega}{2}\sigma_Y t)\exp[i(B\sigma_X - \frac{\omega}{2}\sigma_Y)t]. \tag{30}$$

Having obtained the evolution of the qubit, we can compute the Fisher information for B and ω.

To obtain the optimal quantum Fisher information for the field amplitude B, we calculate

$$\partial_B H(t) = -(\cos\omega t\sigma_X + \sin\omega t\sigma_Z). \tag{31}$$

The optimal quantum Fisher information is determined by the squared gap between the maximum and the minimum eigenvalues of

$$\begin{aligned} h_B(T) =& \int_0^T U^\dagger(0\to t)\partial_B H(t)U(0\to t)\mathrm{d}t \\ =& -\left(\frac{4B^2T}{4B^2+\omega^2} + \frac{\omega^2\sin\left(T\sqrt{4B^2+\omega^2}\right)}{(4B^2+\omega^2)^{3/2}}\right)\sigma_X + 2B\omega\left(\frac{T}{4B^2+\omega^2} - \frac{\sin\left(T\sqrt{4B^2+\omega^2}\right)}{(4B^2+\omega^2)^{3/2}}\right)\sigma_Y \\ & - \frac{\omega\left(1-\cos\left(T\sqrt{4B^2+\omega^2}\right)\right)}{4B^2+\omega^2}\sigma_Z. \end{aligned} \tag{32}$$

From Eq. (2), it can be obtained that the optimal quantum Fisher information for B is

$$I_B^{(Q)} = \frac{16B^2T^2}{4B^2+\omega^2} + 8\omega^2\frac{1-\cos\left(T\sqrt{4B^2+\omega^2}\right)}{(4B^2+\omega^2)^2}. \tag{33}$$

Similarly, to obtain the optimal quantum Fisher information for the field rotation frequency ω, we calculate

$$\partial_\omega H(t) = tB(\sin\omega t\sigma_X - \cos\omega t\sigma_Z). \tag{34}$$

And the h matrix for ω is

$$\begin{aligned} h_\omega(T) =& \int_0^T U^\dagger(0\to t)\partial_\omega H(t)U(0\to t)\mathrm{d}t \\ =& B\left(\frac{\sin\left(T\sqrt{4B^2+\omega^2}\right)}{(4B^2+\omega^2)^{3/2}} - \frac{T\cos\left(T\sqrt{4B^2+\omega^2}\right)}{4B^2+\omega^2}\right)(\omega\sigma_X + 2B\sigma_Y) \\ & + B\left(-\frac{T\sin\left(T\sqrt{4B^2+\omega^2}\right)}{\sqrt{4B^2+\omega^2}} + \frac{1-\cos\left(T\sqrt{4B^2+\omega^2}\right)}{4B^2+\omega^2}\right)\sigma_Z. \end{aligned} \tag{35}$$

And the optimal quantum Fisher information for ω is

$$I_{\omega,0}^{(Q)} = \frac{4B^2T^2}{4B^2+\omega^2} - \frac{8B^2T\sin\left(T\sqrt{4B^2+\omega^2}\right)}{(4B^2+\omega^2)^{3/2}} + \frac{8B^2\left(1-\cos\left(T\sqrt{4B^2+\omega^2}\right)\right)}{(4B^2+\omega^2)^2}. \tag{36}$$

SUPPLEMENTARY NOTE 4. FISHER INFORMATION FOR ω IN THE PRESENCE OF HAMILTONIAN CONTROL WITH ω_c NEAR ω

This Supplementary Note is to obtain the Fisher information for the rotation frequency ω of the magnetic field in the presence of the control Hamiltonian with the control parameter ω_c close to ω.

It has been obtained in the main manuscript that the maximum Fisher information of estimating ω with the optimal Hamiltonian control is

$$I_\omega^{(Q)} = B^2T^4, \tag{37}$$

and the optimal control Hamiltonian is

$$H_c(t) = B(\cos\omega t\sigma_X + \sin\omega t\sigma_Z) - \frac{\omega}{2}\sigma_Y. \tag{38}$$

However, one generally does not know the exact value of ω, so the real control that can be applied on the qubit is

$$H_c(t) = B(\cos\omega_c t\sigma_X + \sin\omega_c t\sigma_Z) - \frac{\omega_c}{2}\sigma_Y, \tag{39}$$

where ω_c is a control parameter that is close to ω, and the maximum Fisher information is saturated when $\omega_c = \omega$. In this case, the total Hamiltonian is

$$H_{tot}(t) = -B(\cos\omega t\sigma_X + \sin\omega t\sigma_Z) + B(\cos\omega_c t\sigma_X + \sin\omega_c t\sigma_Z) - \frac{\omega_c}{2}\sigma_Y. \tag{40}$$

A natural question is how the deviation of ω_c from ω influences the Fisher information. Will a slight deviation of ω_c from ω cause a large drop in Fisher information? This is important to the design and realization of the Hamiltonian control scheme in practice.

In this Supplementary Note, we derive the Fisher information of estimating ω when ω_c is deviated from the true value of ω. We will show how to use this result to design an adaptive control scheme to approach the T^4 scaling of the Fisher information in the next Supplementary Note.

Since the maximum quantum Fisher information of estimating ω is determined by the gap between the highest and lowest levels of $h_\omega(T)$, we need to obtain $h_\omega(T)$ first. As ω_c is close to ω, we can expand $h_\omega(T)$ with ω_c near ω.

First, according to Eq. (10), we have

$$h_\omega(T)\big|_{\omega_c=\omega} = \int_0^T \left[U^\dagger(0\to t)\partial_\omega H_{tot}(t)U(0\to t)\right]_{\omega_c=\omega} dt = -\frac{BT^2}{2}\sigma_Z, \tag{41}$$

where $U(0\to t)$ is the unitary evolution operator under $H_{tot}(t)$, $U(0\to t)|_{\omega_c=\omega} = \exp(i\frac{\omega}{2}t\sigma_Y)$. Also from (10), we can see that

$$\partial_{\omega_c} h_\omega(T)\big|_{\omega_c=\omega} = \int_0^T \left[\partial_{\omega_c}U^\dagger(0\to t)\partial_\omega H_{tot}(t)U(0\to t) + U^\dagger(0\to t)\partial_\omega H_{tot}(t)\partial_{\omega_c}U(0\to t)\right]_{\omega_c=\omega} dt, \tag{42}$$

where we used $\partial_{\omega_c}\partial_\omega H_{tot}(t) = 0$. From Eq. (8), it can be obtained that

$$\begin{aligned}\partial_{\omega_c}U(0\to t)\big|_{\omega_c=\omega} &= -i\int_0^t \left[U(t'\to t)\partial_{\omega_c}H_{tot}(t')U(0\to t')\right]_{\omega_c=\omega} dt' \\ &= \frac{t}{2}\sin\frac{\omega t}{2}(-I + iBt\sigma_X) + i\frac{t}{2}\cos\frac{\omega t}{2}(\sigma_Y - Bt\sigma_Z),\end{aligned} \tag{43}$$

so,

$$\partial_{\omega_c} h_\omega(T)\big|_{\omega_c=\omega} = -\frac{BT^3}{3}\sigma_X. \tag{44}$$

Similarly, according to Eq. (10),

$$\begin{aligned}\partial^2_{\omega_c} h_\omega(T)\big|_{\omega_c=\omega} =& \int_0^T \Big[\partial^2_{\omega_c}U^\dagger(0\to t)\partial_\omega H_{tot}(t)U(0\to t) + 2\partial_{\omega_c}U^\dagger(0\to t)\partial_\omega H_{tot}(t)\partial_{\omega_c}U(0\to t) \\ &+ U^\dagger(0\to t)\partial_\omega H_{tot}(t)\partial^2_{\omega_c}U(0\to t)\Big]_{\omega_c=\omega} dt,\end{aligned} \tag{45}$$

where we again used $\partial_{\omega_c}\partial_\omega H_{\rm tot}(t)=0$. From Eq. (8), it can be seen that

$$\begin{aligned}\partial^2_{\omega_c}U(0\to t)\big|_{\omega_c=\omega}=&-i\int_0^t\Big[\partial_{\omega_c}U(t'\to t)\partial_{\omega_c}H_{\rm tot}(t')U(0\to t')+U(t'\to t)\partial^2_{\omega_c}H_{\rm tot}(t')U(0\to t')\\&+U(t'\to t)\partial_{\omega_c}H_{\rm tot}(t')\partial_{\omega_c}U(0\to t')\Big]_{\omega_c=\omega}{\rm d}t',\end{aligned}\tag{46}$$

and

$$\partial_{\omega_c}U(t'\to t)\big|_{\omega_c=\omega}=-i\int_{t'}^t\Big[U(t''\to t)\partial_{\omega_c}H_{\rm tot}(t'')U(t'\to t'')\Big]_{\omega_c=\omega}{\rm d}t'',\tag{47}$$

so it can be obtained that

$$\partial^2_{\omega_c}U(0\to t)\big|_{\omega_c=\omega}=-\frac{1}{4}T^2(1+B^2T^2)(\cos\frac{\omega T}{2}I+i\sin\frac{\omega T}{2}\sigma_Y)+\frac{i}{6}BT^3(\cos\frac{\omega T}{2}\sigma_X+\sin\frac{\omega T}{2}\sigma_Z),\tag{48}$$

thus,

$$\partial^2_{\omega_c}h_\omega(T)\big|_{\omega_c=\omega}=\frac{4B^2T^5}{15}\sigma_Y+\frac{BT^4}{4}\sigma_Z.\tag{49}$$

Therefore, $h_\omega(T)$ can be expanded in the vicinity of $\omega_c=\omega$ as

$$h_\omega(T)=-\frac{BT^2}{2}\sigma_Z-\frac{BT^3}{3}\sigma_X\delta\omega+\left(\frac{4B^2T^5}{15}\sigma_Y+\frac{BT^4}{4}\sigma_Z\right)\frac{\delta\omega^2}{2}+O(\delta\omega^3),\tag{50}$$

where $\delta\omega=\omega_c-\omega$.

The eigenvalues of $h_\omega(T)$ in Eq. (50) are

$$\lambda_\pm=\pm\frac{BT^2}{2}\mp\frac{1}{72}BT^4\delta\omega^2+O(\delta\omega^4),\tag{51}$$

so, finally, the Fisher information for ω with ω_c near ω is

$$I^{(Q)}_\omega=B^2T^4(1-\frac{1}{18}T^2\delta\omega^2),\tag{52}$$

where higher order terms of $\delta\omega$ have been neglected.

This shows that when ω_c is deviated a little from the true value of ω, the drop in Fisher information is only of the order $\delta\omega^2$, and the T^4 scaling is unaffected, implying the reliability of the above Hamiltonian control scheme. It lays the foundation for the feedback control scheme to approach the T^4 scaling which will be introduced in the next Supplementary Note.

SUPPLEMENTARY NOTE 5. ADAPTIVE HAMILTONIAN CONTROL FOR ESTIMATING ω

Basing on the result in Supplementary Note 4, we can design schemes to approach the T^4 scaling of the Fisher information for estimating ω when the exact value of ω is unknown and an estimate of ω is used in the control Hamiltonian.

We first give an overall analysis about why the Hamiltonian control may increase the Fisher information. In order to apply the Hamiltonian control scheme, we need an estimate of ω to be used as the control parameter ω_c in the control Hamiltonian. But if the precision of the estimate of ω needed is comparable to the precision that can be gained, the Hamiltonian control scheme would be senseless. Fortunately, Eq. (52) shows that an approximate ω with a precision $\delta\omega$ of order $1/T$ is sufficient to produce an estimate of ω with a precision of order T^{-2} (i.e., the Fisher information is of order T^4). This guarantees that the Fisher information can be increased by the Hamiltonian control scheme.

The simplest feedback control scheme is just to first obtain an initial estimate of ω without any Hamiltonian control, then use it in the Hamiltonian control (39) to produce a high precision estimate of ω, without any iteration of the scheme. If the initial estimate of ω without Hamiltonian control is sufficiently good, Eq. (52) guarantees that the final estimate of ω with the Hamiltonian control can attain the Fisher information of order T^4.

For the initial estimation of ω, suppose the qubit evolves for time T without any Hamiltonian control and then it is measured to estimate ω, and this procedure is repeated for N times. According to Eq. (36), the variance of the estimate is

$$\langle \delta\omega^2 \rangle = \frac{4B^2+\omega^2}{4NB^2T^2}, \tag{53}$$

where we assumed $T \gg 1/\sqrt{4B^2+\omega^2}$ to neglect the lower order terms of T for simplicity. Then if we apply the control Hamiltonian with this estimate of ω and let the qubit evolve for the same time T, the Fisher information of the final estimation will be

$$I_\omega^{(Q)} = B^2T^4\Big(1-\frac{4B^2+\omega^2}{72NB^2}\Big). \tag{54}$$

Obviously, if $N \gg \frac{1}{18}(1+\frac{\omega^2}{4B^2})$, then

$$I_\omega^{(Q)} \approx B^2T^4, \tag{55}$$

which reaches the T^4 scaling.

The price for this scheme is that we need additional time NT to obtain an initial estimate of ω in the absence of the Hamiltonian control. But as the additional time NT is linear with T, the T^4 scaling with the Hamiltonian control can still significantly outperform the usual T^2 scaling without the Hamiltonian control.

However, we can also feed the new estimate of ω from the estimation with the Hamiltonian control back into the control Hamiltonian so that a better estimate of ω can be produced, and this step can be iterated to further refine the estimate of ω. This will make the feedback control scheme more efficient.

In the following, we give a detailed analysis about such an adaptive feedback control scheme, and show a minimum requirement for the precision of the initial estimation of ω without Hamiltonian control to make the feedback control scheme work.

For the sake of generality, we assume the Fisher information of each measurement in the initial estimation without Hamiltonian control is I_0, and the measurement is repeated N times, then the variance of the initial estimate of ω is

$$\langle \delta\omega^2 \rangle = \frac{1}{NI_0}. \tag{56}$$

Now, we apply the Hamiltonian control with this estimate of ω and choose the evolution time as $T_1 = \sqrt{I_0}$, then the Fisher information will be

$$I_1 = B^2 I_0^2 (1-\frac{1}{18N}). \tag{57}$$

We repeat the measurement N times, and use the new estimate of ω in the control Hamiltonian. Let the qubit evolve for time $T_2 = \sqrt{I_1}$, then the Fisher information becomes

$$I_2 = B^2 I_1^2 (1-\frac{1}{18N}) = B^6 I_0^4 (1-\frac{1}{18N})^3. \tag{58}$$

If we iterate this process for n rounds and choose the evolution time in the n-th round to be $T_n = \sqrt{I_{n-1}}$, the Fisher information is

$$I_n = B^2 I_{n-1}^2 (1-\frac{1}{18N}). \tag{59}$$

It can be derived from this iterative relation that

$$T_n = I_0^{2^{n-2}} [B^2(1-\frac{1}{18N})]^{2^{n-2}-\frac{1}{2}}, \tag{60}$$

and

$$I_n = I_0^{2^n} [B^2(1-\frac{1}{18N})]^{2^n-1}. \tag{61}$$

In terms of T_n, I_n can be rewritten as

$$I_n = B^2 T_n^4 (1-\frac{1}{18N}). \tag{62}$$

If N is large, $I_n \approx B^2 T_n^4$ then. This recovers the T^4 scaling of the Fisher information in the presence of Hamiltonian control.

A question is how many rounds of feedback control it takes to reach $T_n = T$ for a desired time T. From Eq. (60), it can be obtained that

$$n = \left\lceil \log_2 \frac{\ln\left(BT\sqrt{1-\frac{1}{18N}}\right)}{\ln\left(B^2 I_0 (1-\frac{1}{18N})\right)} \right\rceil + 2. \tag{63}$$

It can be seen that when T is large,

$$n \sim \lceil \log_2 \ln T \rceil, \tag{64}$$

which is a double logarithm of T, implying n increases extremely slowly with T and very few rounds of feedback control is sufficient to reach a large T. So this iterative feedback control scheme converges to the T^4 scaling very fast.

We also want to remark that there is a lower bound that I_0 must satisfy in order to increase the Fisher information by this method. For Eq. (57), I_1 is not always larger than I_0, and if we want to increase the Fisher information, we need $I_1 > I_0$, so it requires

$$I_0 > \frac{1}{B^2(1-\frac{1}{18N})}. \tag{65}$$

When I_0 is above this threshold, I_1 will also be larger than $\frac{1}{B^2(1-\frac{1}{18N})}$ as the Fisher information increases. This again makes $I_2 > I_1$. Repeating this step, we will find that the Fisher information is always increasing, and thus the allowed T for the T^4 scaling is also increasing. On the contrary, if I_0 does not satisfy the lower bound in Eq. (65), I_1 would be smaller than I_0, I_2 would be smaller than I_1, and so forth. Therefore, Eq. (65) is the minimum requirement for the precision of the initial estimation of ω without Hamiltonian control in order to make the iterative feedback control scheme work.

If T is so small that Eq. (63) becomes negative, we just need to set $n = 1$ and the T^4 scaling can be attained. Note that the T_n given in Eq. (60) is just the upper bound of T that the T^4 scaling can be achieved (up to a relative loss of $1/N$) in the n-th round, it does not mean that T must be chosen as T_n in that round.

We summarize this iterative feedback control scheme as follows. First, a rough estimation of ω should be made in the absence of the Hamiltonian control with the Fisher information satisfying the threshold (65), then the estimate of ω is used to apply the Hamiltonian control and get a new estimate of ω. The new estimate of ω can be fed back to the control Hamiltonian to further update the estimate of ω. This procedure is repeated for n rounds until the desired evolution time T is reached, and the number of rounds is given by Eq. (63). The relative loss of Fisher information due to the finite number of measurements is $\frac{1}{18N}$, where N is the number of measurements in each round of the feedback control scheme, which does not affect the T^4 scaling.

SUPPLEMENTARY NOTE 6. ADDITIONAL CONTROL AT LEVEL CROSSINGS OF $\partial_g H_g(t)$

In this Supplementary Note, we analyze the effect of the additional control $H_a(t)$ proposed in the Methods of the main manuscript.

We first prove that under an arbitrary unitary transformation $V(t)$ on the states, a Hamiltonian $H(t)$ is transformed to

$$H'(t) = i(\partial_t V(t))V^\dagger(t) + V(t)H(t)V^\dagger(t). \tag{66}$$

Consider an arbitrary state $|\phi(t)\rangle$ evolving under $H(t)$. It satisfies the Schrödinger equation

$$i\partial_t|\phi(t)\rangle = H(t)|\phi(t)\rangle. \tag{67}$$

Now, if we transform the state $|\phi(t)\rangle$ to $|\varphi(t)\rangle = V(t)|\phi(t)\rangle$, then $|\varphi(t)\rangle$ satisfies

$$\begin{aligned} i\partial_t|\varphi(t)\rangle =& i\partial_t(V(t)|\phi(t)\rangle) \\ =& i(\partial_t V(t))|\phi(t)\rangle + iV(t)|\partial_t\phi(t)\rangle \\ =& i(\partial_t V(t))V^\dagger(t)|\varphi(t)\rangle + V(t)H(t)V^\dagger(t)|\varphi(t)\rangle. \end{aligned} \tag{68}$$

Therefore, the Hamiltonian for $|\varphi(t)\rangle$ is the $H'(t)$ in Eq. (66).

Now we return to our problem. In the presence of the *optimal* control Hamiltonian, the total Hamiltonian is

$$H_{\rm tot}(t) = H_g(t) + H_{\rm c}(t) + H_a(t), \tag{69}$$

where

$$\begin{aligned} H_{\rm c}(t) &= \sum_k f_k(t)|\psi_k(t)\rangle\langle\psi_k(t)| + i\sum_k |\psi_k(t)\rangle\langle\partial_t\psi_k(t)| - H_g(t), \\ H_a(t) &= h(t)[e^{i(\theta_m(t)-\theta_n(t))}|\psi_n(t)\rangle\langle\psi_m(t)| + e^{i(\theta_n(t)-\theta_m(t))}|\psi_m(t)\rangle\langle\psi_n(t)|], \end{aligned} \tag{70}$$

and

$$\theta_k(t) = \int_0^t f_k(t')\mathrm{d}t'. \tag{71}$$

To see the effect of the total Hamiltonian clearly, let us transform the time-dependent eigenbasis $\{e^{-i\theta_k(t)}|\psi_k(t)\rangle\}$ of $\partial_g H_g(t)$ to an arbitrary *time-independent* basis $\{|k\rangle\}$. The transformation can be written as

$$V(t) = \sum_k e^{i\theta_k(t)}|k\rangle\langle\psi_k(t)|. \tag{72}$$

Then the Hamiltonian in the new basis can be obtained from (66).

It is straightforward to verify that

$$i(\partial_t V(t))V^\dagger(t) = i\sum_{kj} e^{i(\theta_k(t)-\theta_j(t))}\langle\partial_t\psi_k(t)|\psi_j(t)\rangle|k\rangle\langle j| - \sum_k f_k(t)|k\rangle\langle k|. \tag{73}$$

and

$$\begin{aligned} V(t)(H_g(t)+H_{\rm c}(t))V^\dagger(t) =& \sum_k f_k(t)|k\rangle\langle k| + i\sum_{kj} e^{i(\theta_k(t)-\theta_j(t))}\langle\psi_k(t)|\partial_t\psi_j(t)\rangle|k\rangle\langle j|, \\ V(t)H_a(t)V^\dagger(t) =& h(t)\sigma_{mn}, \end{aligned} \tag{74}$$

where σ_{mn} is the σ_X-like transition operator between $|m\rangle$ and $|n\rangle$,

$$\sigma_{mn} = |n\rangle\langle m| + |m\rangle\langle n|. \tag{75}$$

So, by the transformation $V(t)$ (72), the total Hamiltonian $H_{\rm tot}(t)$ becomes

$$H'_{\rm tot}(t) = h(t)\sigma_{mn}, \tag{76}$$

where we have used

$$\langle\partial_t\psi_k(t)|\psi_j(t)\rangle + \langle\psi_k(t)|\partial_t\psi_j(t)\rangle = \partial_t\langle\psi_k(t)|\psi_j(t)\rangle = \partial_t\delta_{kj} = 0. \tag{77}$$

Eq. (76) indicates that the state $|n\rangle$ will be transformed to $|m\rangle$ in the new basis $\{|k\rangle\}$ by the total Hamiltonian with the additional control, so in the original basis, $|\psi_n(t)\rangle$ will be transformed to $|\psi_m(t)\rangle$ correspondingly, which is exactly what we want.

Note that $H'_{\rm tot}(t)$ commutes between different t, so the evolution in the new basis $\{|k\rangle\}$ under $H'_{\rm tot}(t)$ for a short time interval $\tau - \frac{1}{2}\delta t \le t \le \tau + \frac{1}{2}\delta t$ is

$$U'(t) = \exp\Big[-i\int_{\tau-\frac{1}{2}\delta t}^{\tau+\frac{1}{2}\delta t} h(t')\mathrm{d}t'\sigma_{mn}\Big] = I\cos\int_{\tau-\frac{1}{2}\delta t}^{\tau+\frac{1}{2}\delta t} h(t')\mathrm{d}t' - i\sigma_{mn}\sin\int_{\tau-\frac{1}{2}\delta t}^{\tau+\frac{1}{2}\delta t} h(t')\mathrm{d}t', \tag{78}$$

where τ is the moment that the level crossing occurs. To drive the system exactly from $|\psi_n(t)\rangle$ to $|\psi_m(t)\rangle$, which corresponds to driving $|n\rangle$ to $|m\rangle$ in the new basis $\{|k\rangle\}$, we need

$$\cos\int_{\tau-\frac{1}{2}\delta t}^{\tau+\frac{1}{2}\delta t} h(t')\mathrm{d}t' = 0, \tag{79}$$

thus $h(t)$ must satisfy

$$\int_{\tau-\frac{1}{2}\delta t}^{\tau+\frac{1}{2}\delta t} h(t')\mathrm{d}t' = \Big(l+\frac{1}{2}\Big)\pi,\ l \in \mathbb{Z}. \tag{80}$$

This proves Eq. (23) in the main text.

A notable point in Eq. (78) is that since $\sin(l+\frac{1}{2})\pi = (-1)^l$, $U'(t)$ will introduce an additional phase $(-1)^{l+1}i$ to $|\psi_m(t)\rangle$ and $|\psi_n(t)\rangle$. This will affect the relative phase and needs to be taken into account, when the system is in a superposed state involving $|\psi_m(t)\rangle$ or $|\psi_n(t)\rangle$.

When there are multiple crossings between the maximum/minimum eigenvalue and other eigenvalues of $\partial_g H_g(t)$ during the whole evolution process $0 \le t \le T$, an additional Hamiltonian control $H_a(t)$ is needed at each level crossing.

SUPPLEMENTARY REFERENCES

[1] Pang, S. & Brun, T. A. Quantum metrology for a general Hamiltonian parameter. *Phys. Rev. A* **90**, 022117 (2014).
[2] Braunstein, S. L. & Caves, C. M. Statistical distance and the geometry of quantum states. *Phys. Rev. Lett.* **72**, 3439-3443 (1994).
[3] Giovannetti, V., Lloyd, S. & Maccone, L. Quantum Metrology. *Phys. Rev. Lett.* **96**, 010401 (2006).
